# Online learning during the Covid-19 pandemic: How university students’ perceptions, engagement, and performance are related to their personal characteristics

**DOI:** 10.1007/s12144-023-04403-9

**Published:** 2023-03-20

**Authors:** Kai Kaspar, Kateryna Burtniak, Marco Rüth

**Affiliations:** Department of Psychology, University of Cologne, Cologne, Germany

**Keywords:** Online learning experiences, Self-regulation skills, Self-efficacy, Big five, Anxiety, Covid-19

## Abstract

University students faced unexpected challenges in online learning during the Covid-19 pandemic. Findings from early phases of the Covid-19 pandemic and before show that online learning experiences may vary from student to student and depend on several personal characteristics. However, the relative importance of different students’ personal characteristics for their online learning experiences at later phases of the Covid-19 pandemic is still unclear. This cross-sectional, correlational study investigates how personal characteristics of university students are related to five dimensions of online learning perception and to their engagement and performance in online courses. In an online survey, 413 students from German universities provided full information on their online learning experiences and personal characteristics in terms of demographic information, Big Five personality traits, self-regulation skills, three facets of self-efficacy, and two types of state anxiety. Results of multiple regression analyses show that students’ age was significantly positively related to all online learning perceptions and engagement in online courses. Our findings also confirm that self-regulation skills and academic and digital media self-efficacy are important factors in various online learning experiences. In contrast, students’ personality traits and state anxiety were less important for most online learning experiences. Noteworthy, several bivariate associations between personal characteristics and online learning experiences are not reflected in the multiple regression model. This underscores the need to consider relevant variables simultaneously to evaluate their relative importance and to identify key personal characteristics. Overall, our results show valuable starting points for theory development and educational interventions.

## Introduction

The sudden change from on-campus to online learning due to the Covid-19 pandemic has disrupted students’ established study routines and social life. Indeed, the Covid-19 pandemic has forced university students to learn online, even in the absence of infrastructural or didactical requirements (e.g., Hoss et al., 2021, 2022; Radu et al., 2020). Consequently, university students have been coping with several technological, educational, and psychological problems related to online learning that have increased since the start of the Covid-19 pandemic (Batdı et al., 2021). How well students adapt to such life changes can heavily rely on their personal characteristics (Caspi & Moffitt, 1993; Pinquart & Silbereisen, 2004). In line with this theoretical assumption, several studies have found that age, gender, and personality traits are related to how strongly people are worried, perceive risks for their life and society, and accept to follow protective measures to counter the Covid-19 pandemic (Kaspar & Nordmeyer, 2022; Zettler et al., 2022). In fact, 56% of the variance in online learning outcomes at the start of the Covid-19 pandemic could be explained based on university students’ personality traits, educational level, and gender (Yu, 2021). Personal characteristics including anxiety were also found to explain a substantial amount of variance (68%) in pre-service teachers’ intention to use digital tools (Rüth et al., 2022). More specifically, university students’ anxiety and personality traits were found to be related to their ability to cope with the transition from on-campus learning to online learning at the start of the Covid-19 pandemic (Besser et al., 2022). So, there is some evidence on the importance of personal characteristics regarding the initial phase of the Covid-19 pandemic (e.g., Besser et al., 2022, Yu, 2021; Zettler et al., 2022), but the role of personal characteristics might be different in later phases of the pandemic beyond the initial emergency phase. Moreover, several studies that examined the importance of personal characteristics for online learning experiences focused on small sets of potentially relevant variables (e.g., Audet et al., 2021; Besser et al., 2022; Yu, 2021). To this end, more personal characteristics may be relevant to students’ online learning experiences, but their relative importance is not yet clear. Hence, a larger set of personal characteristics needs to be considered to unravel their relative importance for university students’ online learning experiences.

Online learning experiences can be manifold, and we here focus on students’ perceptions, engagement, and performance. Indeed, how students perceive digital learning was found to be strongly related to their engagement and performance in digital learning contexts before the Covid-19 pandemic (Rodrigues et al., 2019). Regarding online learning perceptions, there are mixed findings: Students were found to have critical issues with connectivity, accessibility of digital resources, and compatibility of related tools at the start of the Covid-19 pandemic (Agung et al., 2020). Still, survey findings from 2020 based on 1,904 Chilean university students suggest that they experienced online learning in the first semester after the start of the Covid-19 pandemic more positively than they had initially expected, but not as positive as face-to-face education (Lobos et al., 2021). In addition, results of a survey from 2021 indicate that most of the 1,800 U.S. university students surveyed would recommend online learning (83%) and considered learning online to be better than learning on campus (39%) (BestColleges, 2021). These overall positive perceptions could be related to moderate effects of online learning on academic success that were reported in more recent meta-analyses (Ulum, 2022), also when early phases of the Covid-19 pandemic were considered (Batdı et al., 2021). However, it is still unclear which role personal characteristics play in students’ perceptions, engagement, and performance related to online learning at later phases of the Covid-19 pandemic.

To address these research gaps, this correlational study investigates how university students’ online learning experiences were related to a broad set of personal characteristics when the pandemic was more advanced and online learning was no longer in its initial emergency mode.

### Theoretical background and the current study

**Fig. 1:**
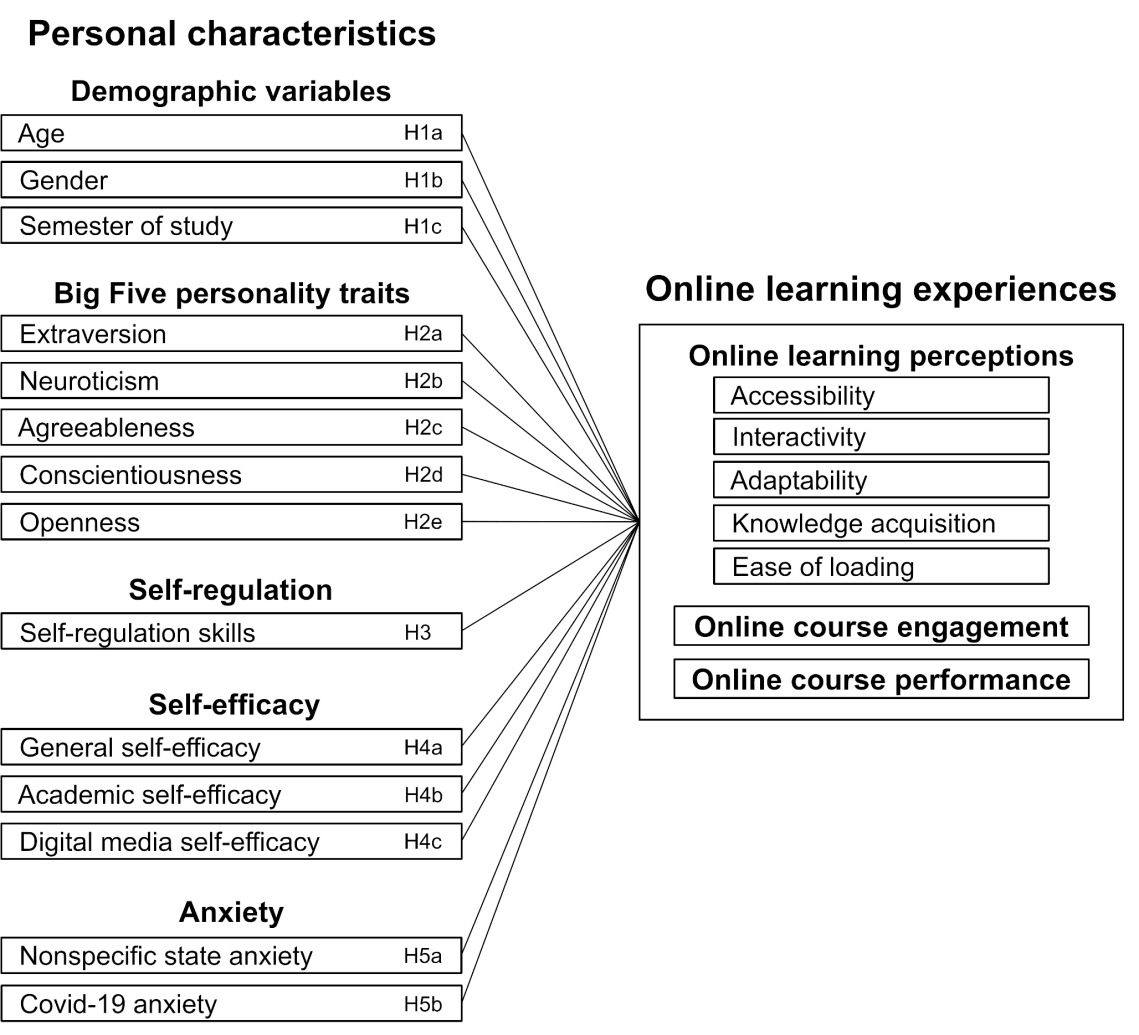
Research model of the present study visualizing the expected relations between students’ personal characteristics and their perceptions, engagement, and performance regarding online learning

In principle, motivation psychology emphasizes that a person’s motivation to strive for a certain goal (such as engaging and performing well in an online learning setting) is modulated by personal and situational factors (Heckhausen, 2020). However, Caspi and Moffitt (1993) stated in their accentuation hypothesis that personal characteristics “are accentuated when environmental events disrupt previously existing social equilibria” (p. 247) and that personal characteristics “should predict behavior best in novel, ambiguous, and uncertain circumstances” (p. 267). This idea fits well with the abrupt and unpredictable changes necessitated by the Covid-19 pandemic, with its particularly strong impact on university students who were used to learning face-to-face on campus and who encountered unprepared infrastructure and missing didactics for online learning under pandemic conditions (see Hoss et al., 2021, 2022). Moreover, that personal characteristics of the individual learner are relevant for online learning is suggested by social cognitive theory with an emphasis on the role of personal agency (Bandura, 2006), by models on the role of self-regulation and self-efficacy (Bradley et al., 2017) as well as personality traits and emotions in online learning environments (Fatahi et al., 2016), and by study results on the role of personality traits in online learning experiences at early phases of the Covid-19 pandemic (Besser et al., 2022; Yu, 2021). Therefore, we assumed that the personal characteristics of university students should show a significant relationship with their perceptions, engagement, and performance under learning conditions in a later phase of the Covid-19 pandemic. However, specific theories modeling the relative importance of personal characteristics for online learning experiences have been lacking. Accordingly, current research in this area is still in an exploratory phase and focuses on identifying particularly relevant factors, often using multiple regression models (e.g., Audet et al., 2021; Besser et al., 2022; Crisci et al., 2021; Jojoa et al., 2021; Yu, 2021). The present correlational study connects here, bringing together five key factors that have been identified as promising candidates in previous studies: demographic variables, the Big Five personality traits, self-regulation, self-efficacy, and anxiety. Figure 1 provides an overview of the research model and a detailed overview of all hypotheses that we derive in the following.

### The role of demographic variables in online learning experiences

Demographic variables that have been frequently studied regarding students’ intention to use digital technology are age, gender, and experience (Venkatesh et al., 2016). Age, gender, and study experience were also found to play a role in students’ online learning experiences (e.g., Diep et al., 2016; Rizvi et al., 2019; Yu, 2021), but results are mixed:

First, age did not play a significant role in online course performance according to some studies (Diep et al., 2016; Ke & Xie, 2009). Still, other studies found that older students showed higher engagement (Chyung, 2007; Ke & Kwak, 2013) and performance in online courses (Dibiase & Kidwai, 2010; Hoskins & van Hooff, 2005; Rizvi et al., 2019). Older students were also found to have better self-regulation skills and to follow a deeper approach to learning (Kizilcec et al., 2017; Richardson, 2013). Still, the role of age needs to be further examined with respect to online learning experiences (cf. Rizvi et al., 2019). In addition, other studies did not examine relations between age and online learning experiences in terms of perceptions, engagement, and performance. Here, we expected that students’ age is positively related to these online learning experiences (H1a).

Second, there are inconsistent findings regarding the role of gender in online learning (e.g., Rizvi et al., 2019; Yu, 2021). Meta-analytic findings suggest that male students have a more positive attitude toward technology use (Cai et al., 2017), but female students were found to be more engaged in online learning (Diep et al., 2016; Shahzad et al., 2021), to achieve higher exam scores than male students in online learning contexts (Chyung, 2007; McSporran & Young, 2001), and to be more satisfied with the first semester of online learning after the start of the Covid-19 pandemic (Lobos et al., 2021). Still, the role of gender in online learning perceptions as well as engagement and performance in online courses is underexplored. Based on previous results, we expected that female students had more positive online learning experiences than male students (H1b).

Third, students’ educational level was found to play a role in online course performance, yet the importance of prior education may decrease throughout course participation (Rizvi et al., 2019). Nevertheless, students with a higher educational degree were found to be less engaged and less satisfied in online learning according to some studies (Diep et al., 2016; Ke & Kwak, 2013), but also more satisfied and successful according to other studies (Li, 2019; Maki & Maki, 2003; Yu, 2021). Overall, study experience can be a relevant factor in online learning, but relations with perceptions, engagement, and performance regarding online learning during the Covid-19 pandemic yet need to be examined. We expected that students’ study experience in terms of their semester of study is related to their online learning experiences (H1c).

### The role of personality traits in online learning experiences

In general, meta-analyses have found significant associations between personality in terms of the Big Five traits (McCrae & Costa, 2008) and academic success, with conscientiousness being the most important factor (Poropat, 2009; Vedel, 2014). More specifically, the Big Five have been related to some online learning experiences, also regarding early phases of the Covid-19 pandemic (cf. Morfaki & Skotis, 2022):

First, students high in extraversion are outgoing and have strong social skills, which was found to be positively related to motivation and satisfaction in online learning (Shih et al., 2013). Extraversion was found to have no relation to online performance (Abe, 2020), yet other studies found a negative relation to students’ exam results in online courses in early phases of the Covid-19 pandemic and before (Maki & Maki, 2003; Rivers, 2021; Yu, 2021). Still, students high in extraversion also showed better adaptability and more positive learning experiences in synchronous online learning at the start of the Covid-19 pandemic (Besser et al., 2022). Overall, we expected significant relations between extraversion and online learning experiences (H2a).

Second, neuroticism was found to be negatively related to students’ academic success (Bahçekapili & Karaman, 2020). Students high in neuroticism tend to experience negative emotions and are more vulnerable to emotional stress, which was related to lower perceived value of online learning (Watjatrakul, 2016) and online learning satisfaction in an early phase of the Covid-19 pandemic (Sahinidis et al., 2020). Students high in neuroticism were also found to adapt worse to the Covid-19 pandemic and to have more negative online learning experiences at the start of the Covid-19 pandemic (Besser et al., 2022). So, we expected that neuroticism is negatively related to online learning experiences (H2b).

Third, agreeableness was found to be positively related to students’ academic success in online learning in early phases of the Covid-19 pandemic (Rivers, 2021; Vlachogianni & Tselios, 2022; Yu, 2021). Agreeable students are polite and willing to compromise and cooperate, and they were found to see more value in online learning for their career (Keller & Karau, 2013). Students high in agreeableness also had high adaptability to the Covid-19 pandemic, which was related to more positive online learning experiences at the start of the Covid-19 pandemic (Besser et al., 2022). Thus, we expected a positive relation between agreeableness and online learning experiences (H2c).

Fourth, conscientiousness was found to be positively related to students’ academic success in online learning (Abe, 2020; Bahçekapili & Karaman, 2020; Rivers, 2021; Yu, 2021). Conscientious students are highly aspired to achieve goals, and it was found that they frequently use learning management systems (Alkış & Temizel, 2018), have positive impressions of online learning (Keller & Karau, 2013; Tavitiyam et al., 2021), and report more satisfaction with online learning in an early phase of the Covid-19 pandemic (Sahinidis et al., 2020). Moreover, students high in conscientiousness could adapt better to the Covid-19 pandemic and had better online learning experiences at the start of the Covid-19 pandemic (Besser et al., 2022). Accordingly, we expected a positive relation between conscientiousness and online learning experiences (H2d).

Finally, openness was found to be positively related to students’ academic success in online learning (Abe, 2020; Bahçekapili & Karaman, 2020; Yu, 2021). Students high in openness are thought to be more creative and to need diverse and novel experiences, which was positively related to students’ perceived value of online learning for their career (Keller & Karau, 2013), as well as to engagement and satisfaction regarding online learning in early phases of the Covid-19 pandemic (Audet et al., 2021; Sahinidis et al., 2020). Openness was also positively related to adaptability to the Covid-19 pandemic as well as to online learning experiences at the start of the Covid-19 pandemic (Besser et al., 2022). Here, we expected a positive relation between openness and online learning experiences (H2e).

### The role of self-regulation skills in online learning experiences

Online learning can make students more aware of self-regulation skills, such as how to plan, control, and evaluate learning processes (Barak et al., 2016). In line with social cognitive theory, self-regulation skills of students were found to contribute to their success in online courses (Bradley et al., 2017; Broadbent & Poon, 2015), and to be positively related to students’ performance in online learning at the start of the Covid-19 pandemic (Anthonysamy, 2021). In this regard, a lack of self-regulation skills was found to be one of the most frequently named problems by students at the start of the Covid-19 pandemic (Hoss et al., 2021). However, relations between self-regulation skills and online learning perceptions as well as engagement and performance in online courses at a later stage of the Covid-19 pandemic are still unclear. We expected that students’ self-regulation skills are positively related to these online learning experiences (H3).

### The role of self-efficacy in online learning experiences

Students high in self-efficacy take constructive approaches in life and believe in their abilities to successfully solve tasks and challenges, as suggested by social cognitive theory (Bandura, 2006). More specifically, students have been dealing with problems related to their academic life, learning online, and the related use of novel digital tools since the start of the Covid-19 pandemic (Batdı et al., 2021). Thus, it seems reasonable that academic and digital media-related abilities have been of particular importance for positive online learning experiences. Accordingly, we considered students’ general self-efficacy, but expected even stronger relations between online learning experiences and students’ academic as well as digital media self-efficacy. Indeed, previous studies indicate the relevance of these three factors: First, students with higher general self-efficacy were found to have more success in online courses (Bahçekapili & Karaman, 2020). Second, several studies reported that academic self-efficacy positively relates to academic success (Honicke & Broadbent, 2016; Rivers, 2021; Yokoyama, 2019). Third, self-efficacy regarding technology and the internet was found to be positively related to online learning perceptions, course satisfaction, and performance in online courses (Bradley et al., 2017; Wang et al., 2013; Wei & Chou, 2020). However, evidence is lacking on the relative importance of general self-efficacy, academic self-efficacy, and digital media self-efficacy for online learning experiences at a later phase of the Covid-19 pandemic. Based on previous results, we expected positive relations between students’ online learning experiences and their general self-efficacy (H4a), academic self-efficacy (H4b), and digital media self-efficacy (H4c).

### The role of anxiety in online learning experiences

Anxiety is a negative emotion that can hamper cognitive performance in terms of lower engagement in tasks and information processing, as suggested by attentional control theory (Eysenck et al., 2007). In line with this assumption, more anxious students were found to have more negative perceptions of online learning and more negative educational experiences at the start of the Covid-19 pandemic (Jojoa et al., 2021; Zhao et al., 2021). Anxiety was higher in students during online learning at the start of the Covid-19 pandemic compared to face-to-face learning (Besser et al., 2022), but anxiety was also found to decrease with experience in online courses (Abdous, 2019). In this regard, it is of particular interest to find out how strongly anxiety is related to online learning experiences in later phases of the pandemic. In addition, it remains unclear whether negative online learning experiences are more strongly related to students’ anxiety in general or regarding the Covid-19 pandemic. Taken together, we examined these relations and expected that students’ online learning experiences are negatively related to their nonspecific state anxiety (H5a) and specific Covid-19 anxiety (H5b).

## Methods

### Participants and procedure

The minimum sample size was *n* = 194 for detecting a significant *R*^*2*^, based on a test power of 0.95, a significance level of 0.05, and a medium-sized effect of *f²* = 0.15. A medium-sized effect was expected based on the outlined theoretical relations between the personal characteristics and online learning experiences as well as on effect sizes from related studies on the role of personal characteristics such as personality traits in academic performance and online learning experiences (e.g., Morfaki & Skotis, 2022; Vedel, 2014). In total, 439 students from German universities participated in this online survey. We excluded 26 people from the analyses because of study at a distance university (*n* = 13), study location abroad (*n* = 9), early termination of the study (*n* = 3), and one person who reported “diverse” as their gender, representing a too small subgroup to be reasonably included in gender-based analyses. The final sample thus consisted of 413 participants (354 female) who were between 18 and 61 years old (*M* = 25.47, *SD* = 7.18). Most participants studied psychology and health-related subjects (community health, human medicine, physiotherapy, and ergotherapy). We invited participants via social media groups aimed at university students by means of convenience sampling and snowball sampling. At the beginning of the study, participants were informed that all students at German universities who were regularly enrolled in the winter semester 2020/2021 and who are at least 18 years old are eligible to participate. They were informed that all data will be collected anonymously and used for research purposes only. Participants who prematurely stopped the survey were not included in the analyses and all their data were deleted. Informed consent to participate in this study was provided by clicking a corresponding box, and participation was voluntary in all cases.

After the participants were informed about the study content and gave their consent, they provided demographic information. They then evaluated their perceptions, engagement, and performance regarding online learning and reported about their personal characteristics (see Fig. 1). The study ran from February 23 to April 26, 2021, so that pandemic-related online teaching and learning was by now an integral part of the study program and beyond the initial emergency phase. Data are available through Open Science Framework (https://osf.io/td5ap/). All instruments in English were translated from English into German and vice versa to ensure that the translated version matches the original version (translation-back translation method) (Brislin, 1970; Maneesriwongul & Dixon, 2004).

### Students’ evaluation of online learning experiences (dependent variables)

#### Online learning perceptions

We used the Online Learning Perception Scale (*OLPS*, Wei & Chou, 2020) to examine five dimensions of online learning perception: *Accessibility* represents students’ perception of free and unlimited access to online materials and resources (e.g., “Online learning provides various multimedia learning resources”, Cronbach’s α = 0.83). *Interactivity* refers to students’ perception of interactions with instructors (i.e., lecturers) and peers in the context of online learning (e.g., “Online learning enables me to interact directly with other learners”, α = 0.85). *Adaptability* refers to students’ perception of their own control over learning time, place, and process in online learning (e.g., “Online learning enables me to decide on the best time to learn”, α = 0.83). *Knowledge acquisition* addresses students’ perception that online learning promotes acquisition of knowledge in desired competence areas (e.g., “Online learning can broaden my common knowledge base”, α = 0.87). *Ease of loading* refers to students’ perception of reduced stress and burdens in online learning (e.g., “Online learning environments are less stressful”, α = 0.87). Content validity was ensured by two researchers with experience in online learning, and the five-factor structure was found via exploratory factor analyses (Wei & Chou, 2020). Online learning perceptions were found to be strongly positively related to students’ online learning readiness in different settings (convergent validity), and weakly related to teachers’ online learning readiness (discriminant validity) (Sarfraz et al., 2022; Wei & Chou, 2020). The 23 items are rated on a scale ranging from 1 (*strongly disagree*) to 5 (*strongly agree*).

#### Online course engagement

Students’ engagement in online courses was assessed by the corresponding subscale of the Online Course Impression instrument (Keller & Karau, 2013). The factorial structure of the instrument was not reported, yet it consists of face-valid items. Empirical findings indicate good construct validity, as student engagement was found to be positively related to conscientiousness, intrinsic motivation, and social presence (convergent validity), and negatively related to amotivation and external regulation (discriminant validity) (Baker & Moyer, 2019; Keller & Karau, 2013). The six items (e.g., “Online courses are very motivating to me” and “I find online courses engaging”, α = 0.84) are rated on a scale ranging from 1 (*strongly disagree*) to 5 (*strongly agree*).

#### Online course performance

Due to the lack of existing measures, we created an instrument measuring perceived performance in online university courses. We considered students’ individual self-evaluation, students’ self-evaluation based on feedback from instructors, and students’ self-evaluation based on feedback from fellow students. While these performance measures are more relevant in ungraded online learning contexts, students were also asked about their course grades considered the status quo performance measure in graded (online) learning contexts (cf. Poropat et al., 2009; Vedel, 2014). Accordingly, participants rated their performance via four items, namely “How would you rate your performance in online courses in general?”, “How would you rate your overall performance in online courses based on feedback from instructors?”, “How would you rate your overall performance in online courses based on student feedback?”, and “How would you rate your performance in online exams based on your grades overall?”. In sum, these items covered student performance in ungraded and graded online courses. Item wording was in German language and the scale’s internal consistency was very good (α = 0.87). The response scale has five steps according to the German grade system (1 = *very good*, 2 = *good*, 3 = *satisfactory*, 4 = *sufficient*, 5 = *poor*). In order to facilitate interpretation of results, the scale was finally inverted so that higher values indicate a better performance.

### Students’ personal characteristics (independent variables)

#### Personality traits

Personality traits were assessed via the short version of the Big Five Inventory (Rammstedt & John, 2005). This is a standardized and economical instrument for applied settings with good construct validity. Its factorial structure was validated in both homogeneous student samples and larger heterogeneous samples using exploratory structural equation modelling (Kovaleva et al., 2013). Moreover, the relations between the five factors and age, gender, and education reported by Kovaleva et al. (2013) were consistent with previous results. The Big Five Inventory comprises 21 items (1 = *disagree strongly*, 5 = *agree strongly*), measuring extraversion (e.g., “I get out of myself, I am sociable”, α = 0.84), neuroticism (e.g., “I get depressed easily, dejected”, α = 0.81), agreeableness (e.g., “I trust others easily, believe in the good in people”, α = 0.65), conscientiousness (e.g., “I complete tasks thoroughly”, α = 0.70), and openness (e.g., “I am interested in many things”, α = 0.71). Personality traits are measured by four to five items each.

#### Self-regulation skills

Self-regulation skills were assessed by the Self-Regulation Scale, an instrument with good predictive validity regarding academic performance and goal commitment as well as good construct validity (Diehl et al., 2006; Luszczynska et al., 2004): self-regulation skills show moderate to strong positive correlations with general self-efficacy, proactive coping, and positive affect (convergent validity), and low to moderate negative correlations with negative affect and depressive symptoms (discriminant validity). The scale comprises ten items (e.g., “I can concentrate on one activity for a long time, if necessary” and “After an interruption, I don’t have any problem resuming my concentrated style of working”, α = 0.84). We used this scale in its original German version (Diehl et al., 2006), with response options ranging from 1 (*not at all true*) to 4 (*completely true*).

#### Self-efficacy

General self-efficacy was measured by means of the validated short general self-efficacy scale (Beierlein et al., 2012). The scale’s content validity was ensured with the help of experts, and its factorial validity was evaluated by means of confirmatory factory analyses. The construct validity of the scale is good in terms of positive associations with another general self-efficacy scale and internal control beliefs (convergent validity), and in terms of negative associations with external control beliefs and neuroticism (discriminant validity). The scale covers three items (e.g., “In difficult situations I can rely on my abilities”, α = 0.83) and uses a 5-point format (1 = *strongly disagree*, 5 = *strongly agree*).

Academic self-efficacy was assessed via seven items of the German Generalized Self-Efficacy Scale (Schwarzer & Jerusalem, 1995) adapted and shortened by Pumptow and Brahm (2021) (e.g., “I face difficulties in my studies calmly because I can rely on my coping abilities”, α = 0.91). Digital media self-efficacy was measured via seven items of the scale for digital media self-efficacy expectation (Pumptow & Brahm, 2021) (e.g., “It’s not difficult for me to reach the objectives I have associated with a media application”, α = 0.96). Both instruments have a unidimensional factorial structure according to exploratory factor analyses and show good construct validity (Pumptow & Brahm, 2021): Academic self-efficacy was found to be positively related to intrinsic motivation (convergent validity) and negatively related to anxiety when studying (discriminant validity); digital media self-efficacy was positively related to usage frequency of digital media and related digital skills (convergent validity), and negatively related to anxiety when studying (discriminant validity). Response scales range from 1 (*strongly disagree*) to 7 (*strongly agree*).

#### Anxiety

Nonspecific state anxiety was rated using the short version of the validated State-Trait Anxiety Inventory that shows good construct validity (Englert et al., 2011): Nonspecific state anxiety was found to be positively related to negative affect (convergent validity), and there was no significant association with positive affect (discriminant validity). Moreover, the factorial validity of the instrument was shown using confirmatory factor analysis. The instrument has five items (e.g., “I am worried that something might go wrong”, α = 0.89) and a response scale ranging from 1 (*not at all*) to 4 (*very*).

Covid-19 anxiety was measured via the validated Coronavirus Anxiety Scale (Lee, 2020a, b). The content validity of the scale was ensured by formulating items that cover clinically relevant symptoms of fear and anxiety, and confirmatory factor analysis supports the scale’s unidimensional factorial structure. Further, there is good construct validity based on positive associations between Covid-19 anxiety and disability, distress, and negative coping mechanisms (convergent validity), and no association with history of anxiety (discriminant validity). The scale consists of five items (e.g., “I felt dizzy, lightheaded, or faint, when I read or listened to news about the coronavirus”, α = 0.85) and uses a response scale ranging from 0 (*not at all*) to 4 (*nearly every day over the last 2 weeks*). The response scale was re-scaled before the analyses for consistency reasons, using a range from 1 to 5.

### Data analysis

First, we examined construct validity in terms of intercorrelations between personal characteristics (independent variables). Second, we analyzed the intercorrelations between online learning experiences (dependent variables). Third, we checked the expected relations between personal characteristics and online learning experiences (H1-H5) by means of multiple regression analyses for each dimension of online learning perception, for online course engagement, and for online course performance. All relevant statistical assumptions concerning multiple regression analysis were met (cf. Poole & O’Farrell, 1971): linearity, normality, and homoscedasticity, but no autocorrelation and no multicollinearity (VIF ≤ 2.61), and we have routinely used bootstrapping for inferential tests as suggested (cf. Hayes & Cai, 2007).

## Results

### Intercorrelations between personal characteristics

As shown by Table 1, intercorrelations between independent variables of the regression models were rather low with few remarkable exceptions (i.e., *r* ≥ .30): Neuroticism was negatively correlated with self-regulation skills (*r* = –.47), general self-efficacy (*r* = –.42) and academic self-efficacy (*r* = –.53), but it was positively correlated with state anxiety (*r* = .53) and Covid-19 anxiety (*r* = .34). In contrast, conscientiousness was positively correlated with self-regulation skills (*r* = .50), general self-efficacy (*r* = .42), and academic self-efficacy (*r* = .39). Self-regulation skills were also positively correlated with general (*r* = .56), academic (*r* = .52), and digital media self-efficacy (*r* = .30), but negatively correlated with state anxiety (*r* = –.48). In addition, general self-efficacy was positively correlated with academic (*r* = .71) and digital media self-efficacy (*r* = .36), but it had a negative correlation with state anxiety (*r* = –.37). Academic self-efficacy had a positive correlation with digital media self-efficacy (*r* = .39) but a negative correlation with state anxiety (*r* = –.43). Finally, state anxiety and Covid-19 anxiety were positively correlated (*r* = .35). Overall, these correlations indicate good construct validity.

**Table 1:** Descriptive statistics and bivariate correlations between independent variables of the regression models

Variable	*M*	*SD*	1	2	3	4	5	6	7	8	9	10	11	12	13
1. Age	25.47	7.18													
2. Gender	n/a	n/a	–0.06												
3. Semester of study	5.80	3.67	0.21***	0.03											
4. Extraversion	3.42	0.92	–0.05	0.06	0.07										
5. Neuroticism	3.33	0.92	–0.08	0.20***	0.07	–0.24***									
6. Agreeableness	3.19	0.82	0.02	0.11*	–0.02	0.22***	–0.14**								
7. Conscientiousness	3.68	0.72	0.03	0.19***	0.16**	0.22***	–0.23***	0.13**							
8. Openness	3.86	0.73	0.12*	–0.01	0.01	0.14**	0.09	0.11*	0.09						
9. Self-regulation skills	2.65	0.49	0.07	–0.02	0.08	0.16**	–0.47***	0.06	0.50***	0.07					
10. General self-efficacy	3.80	0.72	0.06	–0.05	0.15**	0.22***	–0.42***	0.09	0.42***	0.13*	0.56***				
11. Academic self-efficacy	4.64	1.08	0.09	–0.06	0.18***	0.26***	–0.53***	0.14**	0.39***	0.07	0.52***	0.71***			
12. Digital media self-efficacy	4.84	1.34	–0.05	–0.14**	0.08	0.13**	–0.18***	− 0.00	0.16***	0.07	0.30***	0.36***	0.39***		
13. Nonspecific state anxiety	2.06	0.78	–0.08	–0.02	–0.02	–0.24***	0.53***	–0.13**	–0.21***	–0.03	–0.48***	–0.37***	–0.43***	–0.12*	
14. Covid-19 anxiety	1.58	0.69	–0.02	0.10	0.06	0.00	0.34***	–0.10*	–0.06	0.00	–0.22***	–0.18***	–0.25***	–0.12*	0.35***

*Note*. *p < .05, **p < .01, ***p < .001

### Intercorrelations between online learning experiences

As shown by Table 2, intercorrelations between the five dimensions of online learning perception were moderate to high (*r*s ≥ 0.33), and the strongest correlations were between, on the one hand, knowledge acquisition and, on the other hand, accessibility (*r* = .63), interactivity (*r* = .61), and adaptability (*r* = .61). Online course engagement also showed moderate to high correlations with dimensions of online learning perception (*r*s ≥ 0.42) and online course performance (*r* = .39). Finally, online course performance was moderately correlated with knowledge acquisition (*r* = .32), whereas correlations with all other dimensions of online learning perception were only small to moderate (ranging from *r* = .18 to *r* = .26).

**Table 2:** Descriptive statistics and bivariate correlations between dependent variables of the regression models

Variable	*M*	*SD*	1	2	3	4	5	6
1. OLPS: accessibility	3.46	0.88						
2. OLPS: interactivity	2.29	0.83	0.51***					
3. OLPS: adaptability	3.89	0.92	0.47***	0.41***				
4. OLPS: knowledge acquisition	3.04	0.87	0.63***	0.61***	0.61***			
5. OLPS: ease of loading	2.74	1.11	0.33***	0.42***	0.50***	0.50***		
6. Online course engagement	2.65	0.93	0.42***	0.56***	0.44***	0.61***	0.50***	
7. Online course performance	3.64	0.73	0.26***	0.21***	0.21***	0.32***	0.18***	0.39***

*Note*. OLPS = Online Learning Perception Scale; ***p < .001

### Relations between personal characteristics and online learning experiences

The central regression analyses revealed the joint contribution of all personal characteristics to online learning experiences. As shown by Table 3, all personal characteristics explained a significant amount of variance in participants’ online learning perceptions (from 10 to 22% for the five dimensions, all *p* < .001), online course engagement (22%, *p* < .001), and online course performance (29%, *p* < .001). Importantly, when adjusting for multiple testing via Bonferroni correction for all dependent variables (*p* = .007), the results of the corresponding multiple regression analyses (explained variance) remained statistically significant. At the level of individual factors (i.e., independent variables) we found the following pattern of results:

**Table 3:** Results of the multiple regression analyses for online learning perceptions (OLPS), online course engagement, and online course performance

Independent Variables	OLPS: accessibility		OLPS: interactivity		OLPS: adaptability			OLPS: knowledge acquisition		OLPS: ease of loading		Online course engagement		Online course performance	
	β	*p*	β	*p*	β		*p*	β	*p*	β	*p*	β	*p*	β	*p*
Age	.09	.039	.09	.017	.13	< .001		.09	.017	.11	.023	.20	< .001	–.06	.123
Gender	.03	.595	–.02	.619	.10	.045		.01	.858	–.08	.106	–.04	.360	.00	.983
Semester of study	–.09	.071	–.02	.766	–.08	.094		–.03	.485	–.15	.006	–.12	.017	–.02	.599
Extraversion	–.06	.253	–.04	.370	–.12	.025		–.10	.033	–.02	.708	.01	.915	.05	.273
Neuroticism	.16	.037	.11	.123	.10	.110		.15	.035	.09	.209	.18	.008	.20	.003
Agreeableness	–.03	.617	–.01	.824	–.08	.128		–.03	.523	–.09	.081	–.04	.363	–.01	.755
Conscientiousness	.03	.657	–.00	.949	.02	.731		.05	.323	.10	.100	.15	.013	.15	.016
Openness	.05	.397	–.01	.911	.01	.782		.09	.048	.02	.691	.08	.106	.08	.106
Self-regulation skills	.07	.286	.18	.006	.03	.685		.12	.089	.04	.597	.22	.001	.23	.001
General self-efficacy	–.13	.060	–.13	.060	.00	.975		–.06	.399	–.14	.070	–.23	.001	.05	.476
Academic self-efficacy	.19	.010	.10	.192	.13	.070		.17	.028	.16	.061	.18	.007	.28	< .001
Digital media self-efficacy	.24	< .001	.24	< .001	.18	.001		.28	< .001	.11	.045	.23	< .001	.04	.471
Nonspecific state anxiety	–.19	.002	–.17	.008	–.13	.052		–.15	.022	–.17	.013	–.07	.217	–.02	.758
Covid-19 anxiety	–.08	.151	–.02	.795	–.14	.013		–.04	.477	–.02	.756	–.02	.768	–.01	.837
*R* ^*2*^	.17		.15		.15			.22		.10		.22		.29	
*F*	5.74		4.99		5.15			4.97		3.24		8.24		11.74	
*p*	< .001		< .001		< .001			< .001		< .001		< .001		< .001	

*Note.* OLPS = Online Learning Perception Scale; gender was dummy-coded (0 = male, 1 = female); β = standardized regression coefficients of multiple regression analysis and associated *p*-value (bootstrapping via 10,000 iterations, two-tailed)

University students’ age was positively related to all online learning perceptions and online course engagement. In contrast, students in higher semesters of their studies were less engaged in online learning and evaluated the relieving effect of online learning lower (ease of loading). Some associations of age and semester of study were significant in the regression models but not on the level of bivariate correlations between independent and dependent variables (see Table 4). Gender did not show a bivariate correlation with any of the online learning experiences and gender was not a relevant factor in the multiple regression models, except that female (versus male) students perceived more own control over learning time, place, and process in online learning (adaptability).

**Table 4:** Bivariate correlations between independent and dependent variables of the regression models

Independent Variables	OLPS: accessibility		OLPS: interactivity		OLPS: adaptability		OLPS: knowledge acquisition		OLPS: ease of loading		Online course engagement		Online course performance	
	*r*	*p*	*r*	*p*	*r*	*p*	*r*	*p*	*r*	*p*	*r*	*p*	*r*	*p*
Age	.08	.096	.09	.062	.13	.011	.11	.029	.09	.061	.18	< .001	–.03	.539
Gender	.01	.901	–.05	.357	.05	.316	–.02	.716	–.08	.107	–.04	.478	.04	.387
Semester of study	–.02	.711	.04	.416	–.01	.818	.05	.287	–.08	.101	–.00	.937	.09	.084
Extraversion	.01	.909	.02	.733	–.07	.161	–.00	.929	.01	.828	.05	.285	.17	< .001
Neuroticism	–.09	.082	–.11	.023	–.09	.076	–.10	.044	–.11	.031	–.07	.171	–.13	.007
Agreeableness	.01	.907	–.00	.990	–.05	.294	–.01	.902	–.06	.217	–.01	.860	.06	.259
Conscientiousness	.11	.023	.11	.031	.10	.042	.18	< .001	.11	.032	.23	< .001	.37	< .001
Openness	.09	.084	.03	.501	.04	.381	.14	.004	.05	.344	.14	.003	.15	.002
Self-regulation skills	.21	< .001	.26	< .001	.18	< .001	.29	< .001	.16	.002	.29	< .001	.41	< .001
General self-efficacy	.14	.004	.14	.005	.17	< .001	.24	< .001	.08	.091	.13	.008	.39	< .001
Academic self-efficacy	.23	< .001	.20	< .001	.21	< .001	.29	< .001	.16	.001	.22	< .001	.42	< .001
Digital media self-efficacy	.28	< .001	.28	< .001	.22	< .001	.35	< .001	.15	.003	.28	< .001	.24	< .001
Nonspecific state anxiety	–.23	< .001	–.22	< .001	–.19	< .001	–.22	< .001	–.18	< .001	–.16	.002	–.21	< .001
Covid-19 anxiety	–.17	< .001	–.11	.026	–.20	< .001	–.14	.006	–.10	.047	–.08	.095	–.09	.064

*Note*. OLPS = Online Learning Perception Scale; gender was dummy-coded (0 = male, 1 = female)

Overall, students’ personality traits showed only few significant relations to their online learning experiences in the multiple regression models: Extraversion was negatively related to perceived adaptability of online learning and to students’ perception that online learning promotes acquisition of knowledge in desired competence areas (knowledge acquisition). In addition, extraversion showed a positive bivariate correlation with online course performance, but this relation was not significant in the multiple regression model. Neuroticism had negative bivariate correlations with all online learning experiences, these were significant for perceived interactivity with instructors and peers (interactivity), knowledge acquisition, ease of loading, and online course performance. Nevertheless, in the multiple regression models, neuroticism was positively related to students’ perception of free and unlimited access to online materials and resources (accessibility) and knowledge acquisition, as well as to online course engagement and performance. Agreeableness did not show associations with online learning experiences in the regression models and at the level of bivariate correlations. The multiple regression models revealed that conscientiousness was positively related to engagement and performance in online courses. At the level of bivariate correlations, conscientiousness was additionally significantly associated with all dimensions of online learning perception. Openness was only positively related to students’ perception of knowledge acquisition in the multiple regression model and in terms of a bivariate correlation. Additionally, positive bivariate correlations were found between openness and engagement and performance in online courses, but these relations did not reach significance in the regression models.

Students with higher self-regulation skills perceived more interactivity in online learning, and they reported higher engagement and performance in online courses in the multiple regression models. Positive relations to all other online learning perceptions were significant only at the bivariate correlation level. General self-efficacy showed positive bivariate correlations with all dependent variables except ease of loading, yet only the relation with students’ online course engagement was significant and negative in the multiple regression model. The more specific academic self-efficacy and digital media self-efficacy showed positive bivariate correlations with all online learning experiences. A different picture emerged for the multiple regression models: Academic self-efficacy was positively related to students’ perception of accessibility and knowledge acquisition as well as online course engagement and online course performance. Digital media self-efficacy was positively related to all students’ online learning perceptions and to online course engagement. At the same time, digital media self-efficacy was the most relevant independent variable in these domains (except ease of loading), as indicated by the standardized regression coefficients.

Finally, we found negative bivariate correlations between nonspecific state anxiety and all online learning experiences. In the multiple regression models, nonspecific state anxiety was substantially and negatively related to all online learning perceptions, except students’ perception of adaptability. However, no significant relations between nonspecific state anxiety and online course engagement and performance were visible in the multiple regression models. Interestingly, perceived adaptability was the only dimension of online learning perception that was significantly (negatively) related to the more specific Covid-19 anxiety. At the level of bivariate correlations, Covid-19 anxiety showed significant negative correlations with all online learning perceptions, but no significant correlation with online course engagement and performance. Overall, nonspecific state anxiety was rather low (mean value across all participants), and Covid-19 anxiety was even lower (see Table 1).

Notably, Table 4 substantiates the construct validity of the instruments as, for instance, students’ engagement and performance in online courses were positively related to conscientiousness, self-regulation skills, and facets of self-efficacy (convergent validity) and negatively related to nonspecific state anxiety (discriminant validity).

## Discussion

The Covid-19 pandemic has changed university students’ lives worldwide in negative and positive ways (Nowrouzi-Kia et al., 2022). Personal characteristics are thought to relate to how well students can adapt to such changes in their lives, and the focus of this study was to unravel the relations between students’ personal characteristics and their online learning experiences in a later phase of the Covid-19 pandemic when online learning had become an integral part of the study program. In this correlational study, we found that the online learning experiences students had gained over a period of up to one year after the start of the Covid-19 pandemic were significantly related to several of their personal characteristics. The joint consideration of demographic variables, personality traits, self-regulation skills, self-efficacy, and anxiety in the present research model explained a significant amount of variance in all five dimensions of online learning perception (10–22%), in online course engagement (22%), and in online course performance (29%). Thus, we found that a larger set of students’ personal characteristics played a significant role in their online learning experiences. Previous findings suggest that the explanatory value of a reduced model was even greater at the onset of the pandemic – at least with respect to online learning outcomes (Yu, 2021). One explanation for this difference, apart from cross-cultural and other study differences, could be that students have become used to the online learning scenario, so the role of personality characteristics may have diminished as learning conditions became more familiar (cf. Caspi & Moffitt, 1993; Pinquart & Silbereisen, 2004). Overall, our results extend the available evidence on the role of personal characteristics in online learning experiences in terms of perceptions, engagement, and performance.

With respect to the interrelation between students’ online learning experiences, we found moderate to strong associations between perceptions and engagement as well as between engagement and performance. These findings contribute to the ongoing need to better understand the associations between online learning perceptions and engagement (Rodrigues et al., 2019). Moreover, that perceptions and performance showed the weakest (but significant) intercorrelations is in line with previous work that did not find a significant association between online learning perceptions and scores in online courses (Wei & Chou, 2020). Based on this association pattern, we may assume that there is a greater distance between students’ online learning perceptions and self-rated online course performance, whereas self-rated online course engagement is closer to both aspects, reflected in higher correlations. This interesting finding might be a fruitful starting point for modeling the relationship between perceptions, engagement, and performance based on cognitive and behavioral proximity.

Before discussing the results in detail, we highlight three key findings from the multiple regression analyses central to this study, which simultaneously considered the relative importance of several personal characteristics of university students for their online learning experiences.

First, the most frequent positive associations between facets of online learning experiences and personal characteristics occurred with age and digital media self-efficacy, followed by academic self-efficacy and self-regulation skills. These results expand previously found relations between self-regulation skills and self-efficacy for online courses, internet use, and self-regulated learning (Bradley et al., 2017). In line with social cognitive theory, general, academic, and digital media self-efficacy also had strong positive associations with self-regulation skills. Overall, students who report higher self-regulation skills as well as academic and digital media self-efficacy seem to have more positive online learning experiences.

Second, regarding the Big Five personality traits, neuroticism had the most frequent significant positive associations with facets of online learning experiences, and only neuroticism and conscientiousness seem to play a significant role in online course engagement and performance. This expands previous findings by Besser et al. (2022) according to which neuroticism showed the most frequent and strongest relations to several facets of learning experiences in synchronous online learning in the initial phase of the Covid-19 pandemic. However, Besser et al. (2022) also observed many negative correlations between neuroticism and online learning experiences, whereas our regression analyses revealed positive associations between neuroticism and some online learning experiences. Noteworthy, also Audet et al. (2021) and Yu (2021) found some relations between the Big Five traits and facets of online learning experiences that differ from the result pattern of the present study. Importantly, these earlier studies only considered the Big Five or included few additional variables in their multiple regression models. In contrast, the set of personal characteristics was considerably expanded in the present study, so the relative importance of the Big Five must be interpreted on this broader basis. Moreover, the operationalization of the dependent variables differs between studies. Nonetheless, based on the results of the present study, we may conclude that the Big Five traits were of minor importance for university students’ online learning experiences during a later phase of the Covid-19 pandemic.

Third, the most frequent negative associations occurred between the dimensions of online learning perception and nonspecific state anxiety (rather than the more specific Covid-19 anxiety). In addition, we found that university students reported low levels of nonspecific state anxiety and Covid-19 anxiety. Previous work also found that anxiety was low to medium in university students (Besser et al., 2022; Zhao et al., 2021), and our findings show that Covid-19 anxiety was particularly low and of little importance to university students’ online learning experiences in a later phase of the Covid-19 pandemic.

### The relevance of demographic variables for online learning experiences

Our findings suggest that age is a crucial factor that should be considered in online learning, whereas gender and semester of study seem to play a minor role.

First, the multiple regression analyses revealed that students’ age had significant positive relations to all five online learning perceptions. Older (versus younger) students reported more positive perceptions regarding accessibility of learning material, interactivity with others, adaptability of online learning, knowledge acquisition, and ease of loading. Further, older students reported a higher online course engagement, which corroborates previous findings (Cole et al., 2021). Still, age was not associated with higher self-regulation skills, as one might have expected considering the andragogical model of learning (cf. Dibiase & Kidwai, 2010) or since older students were found to have more self-regulation skills and follow a deeper approach to learning (Kizilcec et al., 2017; Richardson, 2013). Moreover, age was not significantly associated with reported online course performance. This contrasts with the finding that older students expected to be less successful in online learning during the pandemic (Hoss et al., 2022), and that age is thought to be an important factor in predicting grades in online course exams (Rizvi et al., 2019). In contrast to the multiple regression models, bivariate correlations between university students’ age and the different facets of online learning experiences were small and non-significant in several cases, indicating the danger of misjudging the relative importance of individual factors in the concert of many factors if only bivariate correlations are used. Overall, we found that older students had more positive online learning perceptions and reported higher engagement in online courses at a later stage of the Covid-19 pandemic.

Second, according to multiple regression analyses, female students reported higher perceived control over learning time, place, and process in online learning (adaptability). In contrast, self-regulation skills showed no significant association with gender, although female students were found to have higher self-regulation skills in previous works on online learning (Alghamdi et al., 2020; Li, 2019). A more general explanation for this relation between perceived adaptability and gender could be that female students were more willing to adapt to changing conditions in the context of the Covid-19 pandemic, as also reflected by higher acceptance and compliance with some protective measures (Kaspar & Nordmeyer, 2022; Zettler et al., 2022). In sum, gender did not play an important role in explaining inter-personal variance in online learning experiences during the Covid-19 pandemic, corroborating previous results (Abdullah et al., 2022; Harvey et al., 2017; Rizvi et al., 2019; Yu, 2021).

Third, semester of study was negatively related to ease of loading and online course engagement in the multiple regression models (but not at the bivariate correlation level). At the same time, older students stated that they had more positive experiences in terms of higher ease of loading and online course engagement. These contrasting findings indicate that it is important to distinguish between students’ age and the number of study-related opportunities to learn. With respect to performance in online courses, semester of study played no significant role. Overall, these results support previous work that reported more experienced students to be less engaged (Diep et al., 2016; Ke & Kwak, 2013), but contradict studies that found a positive association between students’ experience and performance (Li, 2019; Maki & Maki, 2003; Yu, 2021).

### The different roles of personality traits in online learning experiences

The Big Five personality traits were of varying relevance in explaining inter-individual variance in students’ online learning experiences.

First, multiple regression analyses (but not the bivariate correlations) indicate that students high in extraversion perceived reduced own control over learning time, place, and process in online learning (adaptability) and reduced perception that online learning promotes knowledge acquisition in desired competence areas. Previous work indicated that students high in extraversion are able to adapt well to the pandemic and can have positive online learning experiences (Besser et al., 2022). In contrast, the results of the multiple regression model suggest that extraverted students had rather negative online learning perceptions. One explanation for these negative perceptions could be the quality of online learning: extraverted students were found to prefer more group work (Pavalache-Ilie & Cocorada, 2014) and people high in extraversion also reported lower motivation to continue computer-mediated communication in the future at the start of the Covid-19 pandemic (Meier et al., 2021). Accordingly, online learning may not have provided sufficient social interaction in the view of some (more extraverted) students. At the bivariate correlation level, extraversion showed a positive relationship with perceived performance in online courses, but in the multiple regression models, extraversion showed no significant relationship with performance as well as engagement in online courses, which corroborates previous findings (Abe, 2020) but also contrasts other findings (Maki & Maki, 2003; Rivers, 2021; Yu, 2021).

Second, when considered simultaneously with all other personal characteristics in the multiple regression model, students high in neuroticism reported higher engagement and performance in online courses. They also reported a stronger perception that online learning supports knowledge acquisition and that it provides access to free and unlimited learning materials and resources (accessibility). These findings are in contrast with our expectation and with previous results that suggested a negative relation between neuroticism and academic success (Bahçekapili & Karaman, 2020), perceived value of online learning (Watjatrakul, 2016), online learning satisfaction (Sahinidis et al., 2020), and online learning experiences (Besser et al., 2022). One explanation for our findings could be that people high in neuroticism are more engaged in information seeking online (Kaspar & Müller-Jensen, 2021) and that neuroticism is positively related to individuals’ tendency to express their self-aspects in online environments (Seidman, 2013). However, at the bivariate correlation level, neuroticism showed negative correlations with all facets of online learning experiences examined here, although not statistically significant in all cases, indicating that one or more variables in the regression models might have acted as suppressors. This result once again underscores the fact that considering individual variables in isolation from other factors can lead to incorrect assessments of the relative importance of the variables. In this sense, our findings expand previous work that did not find an association between neuroticism and how students evaluate online courses (Keller & Karau, 2013). Previous findings suggested that students high in neuroticism are less able to adapt to the Covid-19 pandemic and are more worried about related consequences (Besser et al., 2022; Zettler et al., 2022). Nevertheless, our findings indicate that higher levels of neuroticism were associated with more positive online learning experiences when other personal characteristics were considered simultaneously (multiple regression model).

Third, we found no significant relation between agreeableness and online learning experiences, neither at the level of bivariate correlations nor in the multiple regression model. By contrast, previous work found positive relations between agreeableness and online learning experiences via adaptability to the Covid-19 pandemic (Besser et al., 2022) as well as students’ engagement in online courses, their perceived value of online learning for their career, and their overall evaluation of online courses (Keller & Karau, 2013). Noteworthy, at the onset of the Covid-19 pandemic, agreeableness had the strongest association of all the Big Five with online learning outcomes in a multiple regression model (Yu, 2021), and was found to play a significant role in online course performance based on path analysis and hierarchical regression (Rivers, 2021; Vlachogianni & Tselios, 2022). Explanations for these differences from our results include that our multiple regression model was more complex in terms of significantly more variables, and that the importance of agreeableness for online learning decreased over the course of the pandemic. In sum, our results put the relative importance of agreeableness into a new perspective.

Fourth and as expected, more conscientious students reported higher engagement and performance in online courses. One explanation could be that people with higher conscientiousness were found to comply more with some Covid-19 restrictions (Krupić et al., 2021), and that better adaptability to the Covid-19 pandemic was positively related to online learning experiences (Besser et al., 2022). More generally, conscientiousness is more related to goal attainment than to compromises and was also found to be consistently related to students’ academic success in online learning (e.g., Abe, 2020; Bahçekapili & Karaman, 2020; Rivers, 2021; Yu, 2021). At the bivariate level, conscientiousness was also significantly positively correlated with all five dimensions of online learning perception. However, conscientiousness was of relatively low importance for online learning perceptions in the multiple regression models, which only partially supports that more conscientious students have more positive impressions of online learning (Keller & Karau, 2013; Tavitiyam et al., 2021). Taken together, our findings support the important role of conscientiousness in positive online learning experiences.

Finally, in the multiple regression model, students with higher openness only perceived higher value in how online learning supports knowledge acquisition. Despite possible differences across courses and universities, one might suspect that students with higher openness also valued the new experiences that online learning offered them, for example, in terms of different digital learning materials and online course designs. Still, we could not confirm the previously found positive associations between openness and online learning engagement (Audet et al., 2021) and online course performance (Abe, 2020; Bahçekapili & Karaman, 2020; Yu, 2021) in the multiple regression model, but we found positive associations at the bivariate correlation level. Importantly, these earlier findings are based on student experiences from the start of the pandemic or before, whereas online learning for students was less of a novel experience in our study. In principle, it could be that openness to (new) experiences as a trait played a more important role in online learning at the beginning of the pandemic than in its later phases. However, this possibility is very difficult to trace based on different cross-sectional studies at different points in time and with different (multiple regression) models. In fact, adding more variables to the model beyond the Big Five traits or replacing variables with other constructs can drastically change the relative importance of each factor because of their complex intercorrelations.

### The benefits of self-regulation for online learning engagement and performance

Independent of a bivariate or multiple regression approach, we found that students with high self-regulation skills perceived online learning to be more interactive and reported higher engagement and performance in online courses. In fact, standardized regression coefficients revealed that self-regulation skills was the second strongest factor regarding engagement and performance in online courses, which corroborates that self-regulation skills are key for successful online learning (cf. Anthonysamy, 2021; Bradley et al., 2017). Self-regulation skills have been conceptualized and operationalized quite differently so far, for example, specifically with reference to self-regulated learning (Panadero, 2017). In contrast, our findings refer to more general self-regulation skills regarding attentional control and goal pursuit (Diehl et al., 2006). Hence, general self-regulation skills are important in the context of online learning, although a causal interpretation of this relationship cannot be derived from the present correlative data.

### The benefits of different facets of self-efficacy for online learning experiences

General, academic, and digital media self-efficacy showed consistently (with only one exception) significant positive correlations with all facets of online learning experiences at the bivariate level. Looking at the multiple regression models, however, the picture was somewhat different: University students’ general self-efficacy was positively associated with engagement in online courses, but this association was negative in the context of the multiple regression model. In this regard, self-efficacy and performance were found to be negatively associated when students were overconfident in their performance in using digital technology (Moores & Chang, 2009). Hence, while our findings are only correlational, one explanation could be that students with high confidence in their abilities show less engagement in online courses.

University students’ performance in online courses was also positively associated with general self-efficacy, but not significantly in case of the multiple regression model. This finding partially contrasts that higher general self-efficacy relates to better performance in online courses (Bahçekapili & Karaman, 2020; Bradley et al., 2017). However, only students’ academic self-efficacy was significantly positively related to perceived performance in online courses, and their digital media self-efficacy was positively related to all five online learning perceptions. These findings emphasize the key role of academic self-efficacy in learning successfully online and on campus (cf. Honicke & Broadbent, 2016; Yokoyama, 2019), and are in line with a positive association between digital media self-efficacy and self-assessed skills in digital learning applications (Pumptow & Brahm, 2021). In addition, these findings illustrate the significance of considering more specific facets of self-efficacy that are directly related to required skills. Overall, academic and digital media self-efficacy are of particular importance for online learning experiences.

### The role of anxiety in online learning perceptions

University students’ anxiety showed negative bivariate correlations with all facets of online learning experiences, being significant in most cases. In contrast, in the multiple regression models, students’ anxiety only played a significant role in explaining online learning perceptions but not online course engagement and performance. Nonspecific state anxiety showed significant negative relations to all dimensions of online learning perception, except adaptability. Adaptability was instead the only online learning experience that was significantly negatively related to the more specific Covid-19 anxiety. Noteworthy, students in our sample had up to a year of experience with online learning in the context of the Covid-19 pandemic, and anxiety was found to decrease with experience in online courses (Abdous, 2019). In sum, anxiety was negatively related to perceptions of online learning but not associated with worse performance in online courses, which is in line with other works (Abe, 2020; Jojoa et al., 2021). Hence, the importance of specific Covid-19 anxiety appears negligible, whereas the role of nonspecific state anxiety in perceptions of online learning could be of more general importance.

### Limitations and future research

Our findings may be associated with some limitations. First, our data are cross-sectional so that experimental and longitudinal research is needed to disentangle causal influences of personal characteristics on online learning experiences. In this regard, personal characteristics may lead to certain experiences and experiences could change personal characteristics (cf. Specht et al., 2011). Second, the available data do not allow to determine whether age itself is a relevant variable or rather other factors that might be related to age, such as underlying differences in using technology or a larger portfolio of general learning strategies. Still, we found that age broadly contributed to explaining several online learning perceptions and online course engagement, and that at least the semester of study and the associated subject-specific knowledge level was not the decisive factor. Third, a distinction could have been made between students who study entirely online and students who take some courses back on campus. However, our focus was to gain an overall picture of online learning during the Covid-19 pandemic. Fourth, regarding our measurement instruments, internal consistency was rather poor for the agreeableness scale (α = 0.65), but good to very good for all other scales (α ≥ 0.70). In addition, our data are based on self-reports, so estimates by third parties (e.g., fellow students or instructors) of students’ actual engagement and performance in online courses could be different. Nevertheless, our results provide important evidence of relevant variables in the context of online learning that could be targeted or, at a minimum, should be considered in terms of different learning trajectories when designing learning opportunities. In this regard, the minor role of personality traits allows future research to limit the focus to the core variables we identified: self-regulation skills, academic self-efficacy, and digital media self-efficacy. Moreover, we may speculate that some of the relations found here are not unique to the Covid-19 pandemic situation. Concerning future research, the amount of explained variance suggests that we missed some constructs that play a role in students’ online learning experiences. For instance, according to the general extended motivational model, personal and situational factors as well as person-situation interactions can motivate or constrain human development and action (Heckhausen, 2020). Moreover, online learning experiences during the Covid-19 pandemic may have included situational, organizational, and interpersonal aspects, such as interactions that are not authentic and of low quality (Niemi & Kousa, 2020; Tzankova et al., 2022). Overall, this exploratory study focused on role of personal characteristics in online learning experiences in terms of explained variance and relative importance, whereas future research could develop and test theoretical assumptions against specific models.

### Practical implications

Attitudes towards online learning have become positive across higher education institutions compared to the start the Covid-19 pandemic and before (Bay View Analytics, 2021; Lobos et al., 2021). Relatedly, infrastructural conditions and educational resources will remain subject to permanent change and technological development (e.g., European Commission, 2022; OECD, 2021). Concerning decisions between online learning and on-campus learning, meta-analytic findings suggest that students who participate in online learning outperform students who learn on campus (e.g., Ebner & Gegenfurtner, 2019; Means et al., 2013). In this context, it will remain crucial to investigate how students perceive and experience the quality of online learning. Hence, the core variables we identified regarding online learning experiences should not only be further investigated but also considered and fostered in practice. Self-regulation skills, academic self-efficacy, and digital media self-efficacy could be addressed, for instance, by means of video lectures, quiz games, or other digital tools for self-study and collaborative learning (e.g., Jansen et al., 2020; Pérez-Álvarez et al., 2018; Rüth et al., 2021). Moreover, training programs were found to have a positive small-to-moderate effect on university students’ self-regulation skills, such as metacognitive, resource management, and cognitive strategies, as well as on academic performance and motivational outcomes (Theobald, 2021). In addition, students’ self-efficacy could be fostered by provision of more elaborate feedback, social interactions in online learning, and motivational mechanisms (Peechapol et al., 2018). In contrast, it seems less effective to consider personal characteristics that were of minor importance in our study, such as to design online learning environments based on students’ gender (cf. Yu, 2021). Instead, our results support taking a more general approach that saves resources in terms of development and distribution of online learning environments (Harvey et al., 2017). In this regard, the overall online learning experience can be improved by considering didactical and technological relations, referencing to theoretical frameworks, and addressing changes and barriers in educational institutions (e.g., Rodrigues et al., 2019; Rüth & Kaspar, 2017).

### Conclusion

Changes in university students’ academic and social life such as the Covid-19 pandemic can emphasize the role of their personal characteristics in coping with these situations. However, the assessment of the relevance of individual characteristics should not take place in isolation from other factors, as the relative importance of the factors only becomes apparent when they are considered simultaneously in basic or advanced research models. Following this approach, we found that the age of students plays a key role in their online learning experiences, with older students having more positive perceptions of online learning and being more engaged in online courses. This suggests putting a stronger focus on the needs, expectations, and experiences of younger students in online learning. In addition, students seem to be more engaged and successful in online courses when they have high self-regulation skills and academic self-efficacy. Engagement in online courses can also be higher when students believe in their abilities related to digital media. In contrast, students’ personality traits played a rather subordinate role in their online learning experiences in a later phase of the Covid-19 pandemic, beyond the initial emergency online learning phase. Moreover, nonspecific state anxiety is negatively related to online learning perceptions. To conclude, online learning has become a common part of higher education, and more positive online learning experiences are related to key malleable personal characteristics of students, specifically self-regulation skills and academic and digital media self-efficacy.

## Data Availability

Data are available through Open Science Framework (https://osf.io/td5ap/).
